# Supplementary material for: Consumer engagement in health care policy, research and services: A systematic review and meta-analysis of methods and effects
Source: PLoS One. 2022 Jan 27;17(1):e0261808. doi: 10.1371/journal.pone.0261808 (PMC8794088; doi:10.1371/journal.pone.0261808)
Supplement: S1 Appendix — (DOCX) [file pone.0261808.s001.docx]

**S1 Appendix. Stakeholder panel members and engagement process**

***Stakeholder panel members***

- Jane Austin, Director, Health Performance Council Secretariat, South Australia
- Allan Ball, Director Consumer and Community Engagement, Women’s and Children’s Health Network, South Australia Director Community Engagement South Australia and Northern Territory, National Disability Insurance Agency, Northern Territory
- Alan Bevan, Health Consumer Representative, South Australia
- Michael Cousins, Manager Community Engagement at Carers SA, South Australia
- Sarah Dalton, Paediatric Emergency Physician, The Children’s Hospital at Westmead; Clinical Executive Director, Agency for Clinical Innovation, New South Wales
- Ellie Hodges, Health Consumer Representative, South Australia
- Lidia Horvat, Manager, Co-design and Consumers, Safer Care Victoria, Victoria
- Ellen Kerrins, Chair SAHMRI Community Advisory Group, South Australia
- Julie Marker, Health Consumer Representative, South Australia
- Michele McKinnon, Executive Director, Quality, Information and Performance, SA Health, South Australia
- Penelope McMillan, Health Consumer Representative, South Australia
- Maria Alejandra Pinero de Plaza, Health Consumer Representative, South Australia; Caring Futures Institute, College of Nursing and Health Sciences, Flinders University, Bedford Park South Australia; National Health and Medical Research Council Transdisciplinary Centre of Research Excellence in Frailty Research to Achieve Healthy Ageing, Adelaide, South Australia
- Judy Smith, Client Liaison Officer, RDNS, South Australia
- David Yeung, Research Fellow, SAHMRI and Haematologist, Royal Adelaide Hospital and SA Pathology, South Australia

***Stakeholder engagement process***

*Advertisement and recruitment of participants:*

Using convenience sampling techniques, potential stakeholder panel members were invited to express interest in participating by circulating details of the project and their proposed role among professionals and consumers, known from within the networks of the researchers. This information was also circulated through different professional forum groups, associations and organisations, including the South Australian Health and Medical Research Institute (SAHMRI) Consumer and Community Engagement Committee members, and the Health Consumers Alliance of South Australia. Potential participants with a range of health professional (e.g. educators, clinicians, policymakers, researchers/guideline developers, service managers) and/or consumer interests (e.g. health consumers, advocates, carers) were eligible to register their interest. Experience of consumer engagement as it related to and within the Australian healthcare context was an essential inclusion criterion. Interested candidates contacted the research team, were provided with an Information Sheet and encouraged to ask questions about the project and their proposed role, and had their participation confirmed as stakeholder panel members through return of a signed consent form.

*Engagement methods, data collection and analysis, and feedback to stakeholders:*

The overall stakeholder approach was modelled on an existing framework.^1^ At key stages of the systematic review process (developing the systematic review protocol, including crafting the research question(s) and population, intervention, comparator/control, outcome [PICO] definitions; initial analyses of results from included studies; draft of the final review report including findings, recommendations and dissemination plan), the stakeholder panel were invited to ‘collaborate’ (IAP2)^2^ and provide their input via face-to-face or telephone semi-structured interviews (protocol development only) and focus group discussions via teleconference facilities. Stakeholder perspectives were specifically sought on the currency and relevance of the original consumer review protocol,^3^ and the appropriateness of interpretations from the study/review findings.

Audiotapes of interview and focus group data were professionally transcribed immediately after each session. In the first instance, checked and corrected semi-structured interview and focus group transcripts were cycled back to each participant for crosschecking and to enhance the credibility of the raw interview data (COREQ item 23).^4^ Responses to each question and discussion point were entered into a Microsoft Excel spreadsheet. Content analyses were used to derive common themes via open coding with two coders who grouped similar responses into categories and assigned labels capturing specific themes. Any discrepancies were discussed among research team members. After consensus was reached in the composition and label of each category, axial coding was used to associate categories to the central phenomena of interest (i.e. perspectives regarding the currency and relevancy of the review protocol, initial analyses of results from included studies, draft of final review report including findings, recommendations and dissemination plan). At each data collection point, summaries of de-identified analysed interview or focus group data were sent to each of the participants to ensure they were satisfied with the ways in which their own data were interpreted and reported (COREQ item 28).^4^ In addition, a document was provided to stakeholders explaining how their feedback had been adopted and embedded into the design, analysis or writing of the review; or the reasons why this was not possible. Majority stakeholder approval was required at each stage of the review, before proceeding to the next review stage could occur.

*References:*

1. Pollock A, Campbell P, Struthers C, Synnot A, Nunn J, Hill S, et al. Development of the ACTIVE framework to describe stakeholder involvement in systematic reviews. J Health Serv Res Pol. 2019;24(4):245-55.
2. International Association for Public Participation Australasia (IAP2). Quality assurance standard for community and stakeholder engagement. Wollongong, New South Wales: International Association for Public Participation Australasia; 2015.
3. Nilsen ES, Myrhaug HT, Johansen M, Oliver S, Oxman AD. Methods of consumer involvement in developing healthcare policy and research, clinical practice guidelines and patient information material. Cochrane Database of Systematic Reviews. 2006(3):CD004563.
4. Tong A, Sainsbury P, Craig J. Consolidated criteria for reporting qualitative research (COREQ): a 32-item checklist for interviews and focus groups. Int J Qual Health C. 2007; 19(6): 349-357.
