# Supplementary material for: Consumer engagement in health care policy, research and services: A systematic review and meta-analysis of methods and effects
Source: PLoS One. 2022 Jan 27;17(1):e0261808. doi: 10.1371/journal.pone.0261808 (PMC8794088; doi:10.1371/journal.pone.0261808)
Supplement: S2 Appendix — (DOCX) [file pone.0261808.s002.docx]

**S2 Appendix.** **Key definitions adopted in the review.**

**Further descriptors for potential outcomes**

**1. Effects on people**

(A) The 'intended recipient participants' of the health care policy, research or services that have been developed, implemented, monitored, or and/or evaluated using the consumer engagement strategy (on whom outcome data may or may not be collected) (i.e. future trial participants, health care service patients, or the wider community)

- Health behaviours (e.g. adherence, compliance, health enhancing life-style or behaviour outcomes, use of interventions or services; and attitudes - added after data extraction)
- Health outcomes (e.g. clinical and physiological measures)
- Knowledge about the resulting products or activities (e.g. participants’ self-rated understanding of a research study from a consent form, or better understanding of health services)
- Satisfaction with the resulting products or activities (e.g. patient feedback ratings on health care services or the use of specific patient feedback in individual staff development)
- Harms or other negative impacts (e.g. complications, morbidity/mortality, relapse, side effects)
- Use or participation (i.e. health care resource utilisation or clinical trial participation).

(B) The 'engagement participants' who are involved in the engagement process (e.g. consumers and/or professionals)

- Satisfaction with the engagement process (e.g. felt valued for expertise and skills)
- Satisfaction with resulting products or activities (e.g. accessibility and responsiveness of health services based on local experience and need)
- Harms or other negative impacts (e.g. feeling threatened by possible reduction of traditional medical-model influence, ‘involvement fatigue’)
- Participation and refusal rates.

We considered assessing outcomes for each of the different types of 'engagement participants' (i.e. health professionals and consumers) to be especially important as there is evidence that health consumers and professionals have different perspectives and experiences of consumer engagement, such as when and how much engagement should occur, its validity, and the challenges of operationalising consumer engagement in practice,^1,2^ which may impact on one of more of our outcomes of interest.

**2. Effects on the research/policy/health care service**

- Quality of the resulting product or activities (examples, compliance of the product or activities with national or international standards
- Relevance of the resulting product or activities (examples, the degree to which subsequently funded research matches patient priorities)
- Uptake of the resulting product or activities (examples, subsequent uptake of product or activity into hospital ward activities).

**3. Process outcomes**

- Consumer perspectives elicited
- Consumer influence on decisions
- Costs and time of the engagement process.

Due to the review topic, we anticipated a range of reported outcomes (and outcome categories) across studies, therefore we did not exclude otherwise eligible studies if they did not report any of these identified outcomes.

*References:*

1. Burns KK, Bellows M, Eigenseher C, Gallivan J. ‘Practical’resources to support patient and family engagement in healthcare decisions: a scoping review. BMC Health Serv Res. 2014;14(1):175.
2. Ward PR, Thompson J, Barber R, Armitage CJ, Boote JD, Cooper CL, et al. Critical perspectives on ‘consumer involvement’ in health research: epistemological dissonance and the know-do gap. Int J Sociol. 2010;46(1):63-82.
