# Supplementary material for: Consumer engagement in health care policy, research and services: A systematic review and meta-analysis of methods and effects
Source: PLoS One. 2022 Jan 27;17(1):e0261808. doi: 10.1371/journal.pone.0261808 (PMC8794088; doi:10.1371/journal.pone.0261808)
Supplement: S3 Appendix — (DOCX) [file pone.0261808.s003.docx]

**Appendix 3. Medline search strategy.**

1. Consumer Participation/

2. Patient Participation/

3. Consumer Advocacy/

4. Consumer Organizations/

5. Public Opinion/

6. ((consumer? or patient? or stakeholder? or user? or lay or citizen? or public or client?) adj (particip$ or involv$ or represent$ or collaborat$ or consult$ or contribut$ or engagement or deliberat$ or dialogue or opinion?)).tw.

7. (citizen$ adj (council? or jury or juries or panel?)).tw.

8. (public adj (meeting? or forum?)).tw.

9. participatory intervention?.tw.

10. ((consumer? or patient?) adj organi#ation?).tw.

11. or/1-10

12. Health Policy/

13. Health Planning/

14. Health Priorities/

15. Policy Making/

16. Decision Making/

17. Decision Making, Organizational/

18. (health$ adj3 (policy or policies or planning or priorit$)).tw.

19. decision making.tw.

20. Health Services Research/

21. Health Care Surveys/

22. Research/

23. participatory research.tw.

24. ((health or health care or health care) adj research).tw.

25. (research adj3 (agenda? or priorit$ or program$)).tw.

26. ((design$ or (recruit$ adj3 subject?) or (data adj3 collect$) or (analysis adj3 data) or dissemination) adj3 (finding? or result? or interviewer?)).tw.

27. research.tw.

28. 26 and 27

29. Guidelines as Topic/

30. Practice Guidelines as Topic/

31. guideline?.tw.

32. Pamphlets/

33. ((health information or information material? or patient information or consumer information or pamphlet? or booklet? or leaflet? or brochure?) adj3 (develop$ or produc$ or evaluat$ or design$ or feed back or feedback or input or in put or comment$)).tw.

34. or/12-25,28-33

35. randomized controlled trial.pt.

36. controlled clinical trial.pt.

37. random$.tw.

38. placebo.ab.

39. trial.ab.

40. groups.ab.

41. or/35-40

42. Animals/

43. Humans/

44. 42 not (42 and 43)

45. comment.pt.

46. editorial.pt.

47. or/45-46

48. 41 not (44 or 47)

49. 11 and 34 and 48
