# Supplementary material for: Consumer engagement in health care policy, research and services: A systematic review and meta-analysis of methods and effects
Source: PLoS One. 2022 Jan 27;17(1):e0261808. doi: 10.1371/journal.pone.0261808 (PMC8794088; doi:10.1371/journal.pone.0261808)
Supplement: Attachment — Submitted filename: PLoSOne_ConsumerEngagement_Review.pdf [file pone.0261808.s005.pdf]

Consumer engagement in health care policy, research, and services: methods and effects

Reviewed for PLoS One
Feb 18, 2021

These authors evaluate controlled trials that examine the potential benefits of engaging consumers in the development of health care services, policy or research. Given the increased focus on including diverse voices in health care policy and systems, this review is timely.

Overall this review is sound and useful. The authors use rigorous criteria for the most part, including registering their trial and using a coherent and sound matrix for classifying interventions and outcomes. I would like to see an Introduction that better framed the contribution, particularly in light of recent efforts to include a diversity of voices and communities in health care versus the extra resource cost of doing so. I also note a potential problem in the meta-analysis across 4 studies in the Results section; authors need to make sure they are not comparing odds ratios to each other across studies.

There are several other minor changes that I noted, which I believe will make the review stronger and more clear. All suggestions are meant to help the authors as they refine this timely work.

Major Revisions:

Authors need to do a better job of explaining the motivation for their work including their definition of consumer engagement. Consumer engagement, at least in the United States, often means engagement and responsibility for one's own medical decisions. Authors note in Appendix S2 that their definition of consumer engagement does not encompass one's own medical decisions, rather they are looking at role of consumers in societal medical decision-making. This definition needs to be stated in Introduction, not in Appendix.

Further, on this same point, authors need to reframe their Introduction to better outline what they hope to learn from this review. Lines 97-99, for example, note that there is already evidence of consumer engagement leading to more accessible and acceptable health services---so why is that studied? The authors also note their main intention is to study "effects"—effects on what? This term is so vague that it is difficult to determine what they are hoping to learn. I was intrigued by the last sentence of the first paragraph, that mentions that intentions around consumer engagement might not live up to the reality of what it is able to achieve, but could not find a description of what exactly those intentions are. (The sentence just prior mentions actual evidence of benefit.) Better organization and framing here would help focus the review.

Lines 250-251: Notes that they contacted study authors in some cases. How often did authors have to contact study authors and what questions were not included in manuscripts? In results, (lines 418-421) authors report that overall risk of bias was high confounded by poor reporting

especially in older studies. More explanation about whether bias risk was due to study design or poor reporting, and how often author contact helped, would help validity of this study.

Results: Lines 444-445: Individual odds ratios across studies are not able to be compared given the different modeling methods and covariates used in individual studies. Thus, the authors cannot conclude that heterogeneity in individual odds ratios represents actual heterogeneity across outcomes. (See Norton, Dowd, Maciejewski; *JAMA.* 2018;320(1):84-85.)

Figures 3/4: It appears odds ratios are being compared across studies in both these figures. It does not appear that odds ratios were compared to generate the overall OR of 0.84 or 1.10, but these comparisons of OR across studies is not valid as explained in citation above. This figure should be removed or modified so as to avoid such comparisons.

Minor Revisions:

Lines 138-146: these lines contain sentence fragments and don't seem to fit well into the flow of the Introduction. Either cut or set up better in preceding text.

Methods: Type of studies. Define what you mean by each of these study types, especially your definition of quasi-RCT

Lines 490-498: This section, "Patient Information" is equivocal in its conclusions, and I think it could be written more definitively. I realized there are only two studies, but not whether outcomes all went the same way, whether there were differences in outcomes between studies, etc. Certainly noting limitation of those conclusions is warranted, but as written now this section is very vague and it's hard to draw anything from it.

Lines 497-498: "Both studies had some unclear risk of bias issues." This sentence and idea need more explanation. What do you mean here? The issues were unclear or the risk of bias was unclear? Unclear how?

Line 517: Sentence beginning "Boivin et al involved consumers…." There is a word missing in this sentence.

Lines 525-527: "There is also some evidence of consumers contributing positively to identifying need and developing mental health services directions, as well as supporting decision-making processes in health." Where is support for this sentence? Is it from Carmen et al study? If so, needs to be made more explicit what outcomes/policies were measured in this study. If not, need citation and possibly study description.

Lines 590-591: given that the neonatal/maternal mortality is one of the primary results, and the only one developed with statistical meta-analysis, more explanation of how the interventions differed between the consumer engagement interventions vs control arms would help. In these interventions, what were some key differences that might have led to better outcomes for the treated group?

> Lines 623-629: I see some more explanation here, but descriptions are very general (e.g. "sustainable community development". More specifics, if you have them, about how these interventions differed would be very helpful for those reading this review to get practical advice on how to develop such interventions.

Lines 678-684: Look again at this section. The pulled quote from the Treweek review is confusing and I had a hard time figuring out how it related to these authors' results.

Lines 693-695: "Health professional can be confident that engaging consumers can have a positive effect in health care policy, research and services, however there are not standard metrics to guide evaluation of this effect." This sentence needs to be changed as it does not have empirical support. If there are no metrics, how do we know effect is positive? Consumer engagement takes additional resources that could be directed to other productive ends. The best use of this section is to help policymakers and clinicians understand the most productive use of consumer engagement, as we know it from existing controlled studies.

Figure 1: In final box of included studies, what is meaning of "plus 5 awaiting classification and 1 ongoing study". Are these included in analysis? I don't think they are but notes in figure and explanation of why they are in the box with final included studies is warranted.
