# Supplementary material for: Consumer engagement in health care policy, research and services: A systematic review and meta-analysis of methods and effects
Source: PLoS One. 2022 Jan 27;17(1):e0261808. doi: 10.1371/journal.pone.0261808 (PMC8794088; doi:10.1371/journal.pone.0261808)
Supplement: Attachment — Submitted filename: R2R_July 2021 updated.docx [file pone.0261808.s006.docx]

**Response to reviewers**

| Peer review comments | Authors’ response |
| --- | --- |
| **EDITOR** |  |
| **21^st^ July 2021**  PONE-D-20-36087R1  Consumer engagement in health care policy, research and services: A systematic review and meta-analysis of methods and effects.  Dr Louise K. Wiles  Dear Dr Wiles,  Thank you for submitting your manuscript entitled "Consumer engagement in health care policy, research and services: A systematic review and meta-analysis of methods and effects." to PLOS ONE. Your manuscript files have been checked in-house but before we can proceed we need you to address the following issues:  1. Thank you for revising your manuscript according to our requests and apologies for the delays you have experienced while we assess your manuscript. As a member of Committee on Publication Ethics (COPE), PLOS ONE takes seriously all cases of potentially inappropriate text re-use and overlap.  We appreciate that your study describes an update of the systematic review performed by Nilsen et al. (https://www.cochranelibrary.com/cdsr/doi/10.1002/14651858.CD004563.pub2/full/es), and that some degree of overlap in the Methods may be inevitable. However, we note that there is still significant text overlap between your submission and Nilsen et al. in the reporting of the Results. Specifically, we noted overlap between lines 689-709 of your submission, and the first four paragraphs of the section “Consumer involvement versus no consumer involvement in research” of the Nilsen et al article. Since this section of your manuscript is a narrative description of the results, we request that you please revise the manuscript to rephrase the duplicated text and provide details as to how the current manuscript advances on previous work.  We also note that there is significant overlap between lines 721-724 and the final subheading on page 22 of the following source: https://www.who.int/evidence/sure/PatientSafetyfullreport10052014.pdf?ua=1. Please revise the manuscript to rephrase the duplicated text and cite your sources.  We hope you understand the reasons for this request, and look forward to hearing from you.  Your manuscript has been returned to your account. Please log on to PLOS Editorial Manager at https://www.editorialmanager.com/pone/ to access your manuscript.  Your manuscript can be found in the "Revisions Sent Back to the Author" link under the New Submissions menu. After you have made the changes requested above, please be sure to view and approve the revised PDF after rebuilding the PDF to complete the resubmission process.  Please note that these changes have been requested to comply with submission guidelines and your manuscript will *not* be sent to review until you have fully adhered to our requests. Once your paper has been seen by an Editor we may return it to you for further information or amendments.  We ask that you address this request within 21 days. If you require additional time, please email the journal office. We are happy to grant extensions of up to one month past this due date. If we have not heard from you within 21 days, your manuscript will be withdrawn from Editorial Manager.  Thank you for submitting your work to PLOS ONE.  Kind regards,  Samantha Russell  PLOS ONE | Thank you again for the opportunity to revise our manuscript. We appreciate and understand the reasons for the requests you’ve outlined in our correspondence, and we are happy to address these as detailed below. We are complete agreement that these issues need careful attention.  Section 689-709  We have added in sentences to clarify that this section is largely similar to Nilsen et al and only differs with the addition of one study – and that this makes no difference to the results from the earlier review. We have significantly rewritten the sentences and deleted extraneous points.  Section 721-724  This duplication appears serendipitous. However we have re-written the sentence:  They found no overall difference in understanding between the two groups.  From the original: Overall, there were no statistically significant differences between trial groups for understanding of the parent trial at any time point for any of participant characteristics. |
| **21^st^ June 2021**  PONE-D-20-36087R1 Consumer engagement in health care policy, research and services: A systematic review and meta-analysis of methods and effects. PLOS ONE  Dear Dr Wiles,  Please address the following lines in the Introduction, Results, and Discussion section:   - Lines 96 – 105 - Lines 146 – 156 - Lines 188 – 194 - Lines 674 – 683 - Lines 687 – 707 - Lines 719 – 722 - Lines 755 - 862   Please submit your revised manuscript by July 9th. If you need additional time, please let us know.   Regards, Anita Estes PLOS ONE | Thank you – we have addressed each of these below by line sections. The original text is presented in the left-hand column, and our revised text (with explanations/justifications) on the right.  Please note: some of the line numbers of the revised text have changed in response to our edits. |
| **Lines 96-105 – original text**  *“…The concept of consumer engagement is founded on the principle that health care policy, research and health services are in the public interest [1] and people have a moral right to contribute to decisions which will affect them and have been publicly resourced through government funds and charitable donations [1, 2]. In health policy, input from consumers is typically sought to promote democratic accountability, to ensure that diverse and divergent perspectives are introduced into policy discussions, and to test the suitability of different deliberative techniques to generate evidence for policymaking [3]. In health research, evidence supports the notion that consumer engagement leads to research of greater quality and clinical relevance [4] and greater uptake of findings [5]. There is also some evidence that input from consumers in planning health care can lead to more accessible and acceptable health services [6]…”* | Lines 91-100 – revised text  In response, changes have been made to the text. The cited references were checked, and some wording changes were made to ensure the concept was unchanged and still discussed, but that the wording was unique (notably, it was acknowledged this was the case for reference [3]).  *“…The concept of consumer engagement is founded on the principle that health care policy, research and health services are in the public interest [1] and people have the right to be engaged with and contribute to decisions which will affect them [1, 2]. In their review, Degeling et al [3] found the purpose for involving consumers in health policy processes is to capture the plethora of community perspectives, to enable consumer responsibility , and to examine acceptability of approaches to generate evidence for policymaking [3]. In health research, evidence supports the notion that consumer engagement leads to research of greater quality and clinical relevance [4] and application of findings [5]. There is also some evidence that input from consumers in planning health care can lead to more accessible and acceptable health services [6]…”* |
| **Lines 146-156 – original text**  *“…In our review, we aim to report on: (a) the methods of consumer engagement strategies used in societal decisions on health care policy, research and services (according to the IAP2 Public Participation Spectrum); and, (b) their effects on the people involved in the engagement, on the research/policy/health care service, as well as process outcomes.*  *The primary objective of the review was to assess the effects of consumer engagement on health care policy, research and services. Secondary objectives were to explore whether differences between studies might explain any differences between the effects. We were specifically interested in differences in the:*   - *methods (levels) of consumer engagement (e.g. fact sheets, focus groups, patient advisory committees representing the levels of inform, consult, involve, collaborate or empower);* - *stages (i.e. development, implementation, monitoring, evaluation) of health care policy, research and services in which consumers are engaged; and* - *characteristics of consumer or professional participants (e.g. background, experience or training in consumer engagement)…”* | Lines 138-154 – revised text  In response and to provide further clarity we have added *“In our review which updates the 2009 review [23]…”* at line 145 (now, lines 138-139). We have also cited this reference at additional points within this passage of text.  In response to lines 146-156, these outline our primary and secondary objectives, which formed the basis of our search and review which cannot be altered. No further changes can be made, as our review is an update of the sources that you have provided, of which we have appropriately cited. It is acknowledged they overlap with your provided sources of:   - <https://cccrg.cochrane.org/priority-reviews/more-about-our-consumer-engagement-priority-review> - this source is advising what this updated review will be about; AND - <https://doi.org/10.1002/14651858.CD004563.pub2> - this is the review that we are updating and have cited as [23].   “…In our review which updates the 2009 review [23], we aim to report on: (a) the methods and effects of consumer engagement strategies used in societal decisions on health care policy, research and services (according to the IAP2 Public Participation Spectrum); and, (b) their effects on the people involved in the engagement, on the research/policy/health care service, as well as process outcomes.  The primary objective of the review was to assess the effects of consumer engagement on health care policy, research and services [23].  Secondary objectives were to explore whether differences between studies might explain any differences between the effects [23]. We were specifically interested in differences in the:  • methods (levels) of consumer engagement (e.g. fact sheets, focus groups, patient advisory committees representing the levels of inform, consult, involve, collaborate or empower) [23];  • stages (i.e. development, implementation, monitoring, evaluation) of health care policy, research and services in which consumers are engaged [23]; and  • characteristics of consumer or professional participants (e.g. background, experience or training in consumer engagement) [23]…” |
| **Lines 188-194 – original text**  *“…We used the following definition of health care consumer: patients and potential patients, carers, and people who use health care services. Collectively, ‘consumers’ and ‘community members’ may be referred to as ‘the public’ [32]. However, given the variations in terminology within different contexts, we included any of the following terms for health care consumers: patients; unpaid carers (current or former); parents/ guardians/family; users and potential users of health care services; people with lived experience; peer workforce; people with disability; members of the public who are the potential recipients of…”* | Lines 183-189 – revised text  These lines of text outlined a definition we used as the basis of our search. Our definition was informed by other references to ensure it was based on current and best evidence, which have been duly cited (please see below).  Of these different terminologies we used, we acknowledge that there is some overlap with the terms in references you provided as source documents, however: (1) they represent common terms and synonyms of terms and therefore cannot be entirely unique, and (2) we are not able to change our definition post hoc, especially as this definition informed the search strategy we employed. In light of these reasons, we have not made changes to this passage of text – however are happy to hear from and work with the Editorial Team on any further rewording suggestions should they be desired.  *“…We used the following definition of health care consumer: patients and potential patients, carers, and people who use health care services. Collectively, ‘consumers’ and ‘community members’ may be referred to as ‘the public’ [32]. However, given the variations in terminology within different contexts, we included any of the following terms for health care consumers: patients; unpaid carers (current or former); parents/ guardians/family; users and potential users of health care services; people with lived experience; peer workforce; people with disability; members of the public who are the potential recipients of…”* |
| **Lines 674-683 – original text**  *“…Carman et al [64] collaborated with consumers in exploring ethical decision-making in health care - they compared different ways of deliberating with the consumer group via face to face or online options compared to no consumer engagement and reading material only, and found that all options which involved consumer engagement were superior to the control group for knowledge and attitudes towards decision-making, as well as attitudes to hospital use. Based on these studies there is some evidence that consumers may have a role in identifying a broader range of health care priorities that are complementary to those identified by professionals [45, 54]. In addition to supporting decision-making processes in health (reflected in public deliberation increasing participants’ knowledge of and attitudes towards the role of medical evidence) [64], there is also some evidence of consumers contributing positively to identifying need and developing mental health service directions [53]….”* | Lines 658-667 – revised text  We have reviewed our cited references and the references provided as source documents for cross-checking and have not been able to identify any wording overlap with our text. No changes have been made, however we are happy to hear from and work with the Editorial Team on any further rewording suggestions should they be desired.  *“…Carman et al [64] collaborated with consumers in exploring ethical decision-making in health care - they compared different ways of deliberating with the consumer group via face to face or online options compared to no consumer engagement and reading material only, and found that all options which involved consumer engagement were superior to the control group for knowledge and attitudes towards decision-making, as well as attitudes to hospital use. Based on these studies there is some evidence that consumers may have a role in identifying a broader range of health care priorities that are complementary to those identified by professionals [45, 54]. In addition to supporting decision-making processes in health (reflected in public deliberation increasing participants’ knowledge of and attitudes towards the role of medical*) *[64], there is also some evidence of consumers evidence contributing positively to identifying need and developing mental health service directions [53]…”* |
| **Lines 687-707 – original text**  *“…The fourth policy-based trial [53] collaborated with consumers with previous mental health issues in identification, planning, development (and implementation) of community-based services. The services developed with consumer collaboration were superior to services developed with no consumer engagement in all measured items (mental health scores, service use and so forth), except employment and medication use.*  *The remaining policy-related trial [11] convened two parallel guideline development groups, one with and one without consumers, to craft Population, Intervention, Comparator, Outcome, Time (PICOT) questions, and identify key benefits and harms on the topic of using amyloid positron emission tomography in the diagnosis of dementia. The proposed guideline questions, and benefits and harms, were largely similar between the two groups; however, only the group that collaborated with consumers proposed outcomes around the future development of cognitive impairment at certain time points and proposed rate of progression (rather than considering the development of dementia as a binary [yes/no] outcome). Consumer collaboration also influenced the conduct of guideline development, scope, inclusion of patient-relevant topics, outcome selection, and planned approaches to recommendation development, implementation, and dissemination…”* | Lines 667-685 – revised text  In response, direct quotation marks and page reference numbers have been added to the Armstrong [11] summary; this is due to the direct overlap of text used and inability to alter the wording without changing the meaning and inferences of the reported study results and findings.  *“…The fourth policy-based trial [53] collaborated with consumers with previous mental health issues in identification, planning, development (and implementation) of community-based services. The services developed with consumer collaboration were superior to services developed with no consumer engagement in all measured items (mental health scores, service use and so forth), except employment and medication use.*  *The remaining policy-related trial [11] compared two guideline development groups, one collaborating with consumers and one without, to craft Population, Intervention, Comparator, Outcome, Time (PICOT) questions, and identify key benefits and harms on the topic of using amyloid positron emission tomography in the diagnosis of dementia. “The proposed guideline questions, and benefits and harms, were largely similar* *between the two groups” [11 p.1]; however, only the group that collaborated with consumers “proposed outcomes around the future development of cognitive impairment at certain time points and proposed rate of progression (rather than considering the development of dementia as a binary [yes/no] outcome) [11 p.9-10]. Armstrong et al [11] reported that consumer collaboration also influenced the “conduct of guideline development, scope, inclusion of patient-relevant topics, outcome selection, and planned approaches to recommendation development, implementation, and dissemination” [11 p.11]…”* |
| **Lines 719-722 – original text**  *“…In this trial the average satisfaction score was a little lower in the consumer (client) interviewed group than it was in the staff interviewed group (0.16 on a scale from 1 to 4, p = 0.05). When the results of these two trials [59,60] were pooled, the overall difference was similar (0.14 on a scale from 0 to 4, p = 0.001), (Fig 5)…”* | Lines 700-704 – revised text  This text represents a result from a study; we are unable to alter the wording as it is already so concise (stating the outcome measure and score) and wording changes would alter the meaning. In response and to ensure the study is adequately referenced, we have now added an additional citation number.  *“…In this trial the average satisfaction score was a little lower in the consumer (client) interviewed group than it was in the staff interviewed group (0.16 on a scale from 1 to 4, p = 0.05) [60]. When the results of these two trials [59,60] were pooled, the overall difference was similar (0.14 on a scale from 0 to 4, p = 0.001), (Fig 5)…”* |
| **Lines 755-862 – original text**  *“…pregnant women can reduce neonatal mortality, although there was no effect on maternal death rates. Coupled with other single trials, there is emerging evidence that consumer engagement in health service delivery affords benefits (and no evidence of harms to date) and these seem to be particularly evident in community health care settings. It should also be noted that the outcomes of the health services trials were wide ranging beyond health effects on the intended trial participants and included satisfaction, health behaviour adoption, knowledge and service utilisation. There is some evidence that consumers can have a positive role in the training of health professionals and in providing information for patients to inform their decision-making, although there is still no trial evidence for the role of consumers in guideline production.*  *Investigations into the effect of consumer engagement in health policy were less prevalent but did include some trials demonstrating potential benefits of collaborating with consumers in health priority-setting where they can broaden the perspectives otherwise gained from health professionals alone. There remains a dearth of investigation into the effect of consumer engagement in policy decision-making. The investigations of the effect of consumer engagement in health research remains limited to process indicators like recruitment, adherence, information and satisfaction, with no trials investigating the benefits of consumers being engaged in setting a research agenda or research methodology.*  *Secondary objectives were to explore whether differences between studies might explain any differences between the effects. We were specifically interested in differences in the:*   - *methods (levels) of consumer engagement (e.g. fact sheets, focus groups, patient advisory committees);* - *stages (i.e. development, implementation, monitoring, evaluation) of health care policy, research and services in which consumers are engaged; and* - *characteristics of consumer or professional participants (e.g. background, experience or training in consumer engagement).*   *Such a broad range of trials did not allow for rigorous investigation of the influence of these differences using sub-group analyses. However, there are patterns that warrant commentary. Firstly the strongest evidence (community-based antenatal care for pregnant women) lay in the* ***method of engagement*** *that lies within the definition of empowerment - using the definitions from the IAP2 of placing "the final decision-making in the hands of the public" and with the tacit understanding "we will implement what you decide" [28]. The four studies [44, 47, 50, 51] that provided the evidence for improved neonatal mortality all empowered consumers in this decision-making, with the consumer supported project and sustainable community development. Other key characteristics were that the local community (i.e. women’s groups, community leaders and member, locally recruited facilitators) were embedded in every phase of the CE program; in developing its content, its implementation plan, and its delivery. The majority of the remaining studies used collaborative methods such as advisory groups, special meetings and committees; and smaller numbers using involving methods (via public meetings) or consulting (via focus groups and surveys), Table 1 and Table 2.*  *The predominant* ***stage of consumer engagement*** *was in development (over 75%), with a third of these [44, 47, 50, 51, 53, 62] (including the four comprehensive health service interventions for healthy birthing) [44, 47, 50, 51] undertaking engagement in both development AND implementation. This seems a logical paired process and again was most common in the community settings, to ensure buy-in and relevance for service delivery in most examples. Only one trial [52] collaborated with consumers at all stages from development through to evaluation. However, the numbers are too small to allow a statistical analysis of the import of this factor on effectiveness.*  *Finally, we considered the* ***characteristics of the consumers*** *in the trials. Again, there was a consistent finding that the consumers were not formally trained in engagement methods prior to the engagement, but the majority received requisite training in the study methodology or intervention to be delivered as part of the engagement process. In three studies [52, 59, 60], health professional participants were reported to receive this same training. Only eight trial consumer cohorts [11, 49, 54, 55, 58, 61, 62, 64] received no training before or during the trial - bringing their lived experience as sufficient expertise for the purposes of the evaluation. This is an important finding for future trials - to consider the engagement of all participants – professionals and consumers – in health care stages across policy, research and services.*  *We found no evidence of adverse effects in engaging with consumers; most trials did not collect data on this aspect. The trials with patient health level data (such as neonatal mortality) [44,50] considered unintended effects in terms of examining the risk ratios for all outcomes between the two groups. None of the included trials addressed possible other adverse effects of consumer engagement, such as tokenism or time and other resource impacts. Seven studies [43,45-47, 50 ,51,53] did consider cost-effectiveness, reporting favourably on the cost per life saved and/or cost of years of life saved in the case of the neonatal care studies, or the cost of the intervention itself, but with no benefits or effectiveness analysis [53].*  ***Strengths and weaknesses of the study***  *By including a strong stakeholder engagement at key stages in the review process we believe we have mitigated against any major biases.*  *Our included trials were wide ranging internationally and across diverse health populations. In particular, we were struck by the number of trials engaging with consumers who were most likely to experience disparity or disadvantage in health services. The settings were predominantly in the community - this seems to lend itself to participatory processes. However, there were some examples of consumer engagement in acute and outpatient settings that augur well for ongoing investigations across the health services spectrum. Positive findings across this range suggests that consumer engagement has a role in many settings, however this does not negate the need for consideration to always be paid to these contextual factors when developing, implementing, monitoring and evaluating consumer engagement processes.*  *Whilst we note the increase in number of more strongly designed (randomised controlled) trials in this important area, most still had a relatively high risk of bias. It should be acknowledged that it can be hard to blind participants – consumers and professionals – in a health care intervention. However, more achievable aspects like adequate randomisation and allocation should be more stringently adhered to, as well as better reporting of outcomes and attrition.*  ***Strengths and weaknesses in relation to other studies***  *Treweek et al [65] published a Cochrane review regarding strategies to improve recruitment to randomised trials. Their key results relating to consumer engagement in recruitment are similar to the results we have found with respect to patient information; in that consumer involvement in developing the content, format and appearance of information leaflets for potential research participants made little or no difference to recruitment (absolute improvement was 1%, 95% CI: −1% to 3%) [65]…”* | This passage of text contains our:   (a) objectives – the wording of which which cannot be altered post hoc, and (b) stated strengths and weakness – which reflect our own opinions, and as such, we have not been able to identify how these overlap with the source documents provided.   While we have made some minor changes in response, more substantial edits were not possible given these reasons (i.e. objectives are to remain unchanged, and no overlapping text with sources identified). We have also checked to ensure that citations to the relevant sources (i.e. studies, and their results/findings) have been provided.  *“…pregnant women can reduce neonatal mortality, although there was no effect on maternal death rates. Coupled with other single trials, there is emerging evidence that consumer engagement in health service delivery affords benefits (and no evidence of harms to date) and these seem to be particularly evident in community health care settings. It should also be noted that the outcomes of the health services trials were wide ranging beyond health effects on the intended trial participants and included satisfaction, health behaviour adoption, knowledge and service utilisation. There is some evidence that consumers can have a positive role in the training of health professionals and in providing information for patients to inform their decision-making, although there is still no trial evidence for the role of consumers in guideline production.*  *Investigations into the effect of consumer engagement in health policy were less prevalent but did include some trials demonstrating potential benefits of collaborating with consumers in health priority-setting where they can broaden the perspectives otherwise gained from health professionals alone. There remains a dearth of investigation into the effect of consumer engagement in policy decision-making. The investigations of the effect of consumer engagement in health research remains limited to process indicators like recruitment, adherence, information and satisfaction, with no trials investigating the benefits of consumers being engaged in setting a research agenda or research methodology.*  *Secondary objectives were to explore whether differences between studies might explain any differences between the effects. We were specifically interested in differences in the:*   - *methods (levels) of consumer engagement (e.g. fact sheets, focus groups, patient advisory committees);* - *stages (i.e. development, implementation, monitoring, evaluation) of health care policy, research and services in which consumers are engaged; and* - *characteristics of consumer or professional participants (e.g. background, experience or training in consumer engagement).*   *Such a broad range of trials did not allow for rigorous investigation of the influence of these differences using sub-group analyses. However, there are patterns that warrant commentary. Firstly the strongest evidence (community-based antenatal care for pregnant women) lay in the* ***method of engagement*** *that lies within the definition of empowerment - using the definitions from the IAP2 of placing "the final decision-making in the hands of the public" and with the tacit understanding "we will implement what you decide" [28]. The four studies [44, 47, 50, 51] that provided the evidence for improved neonatal mortality all empowered consumers in this decision-making, with the consumer supported project and sustainable community development. Other key characteristics were that the local community (i.e. women’s groups, community leaders and member, locally recruited facilitators) were embedded in every phase of the CE program; in developing its content, its implementation plan, and its delivery. The majority of the remaining studies used collaborative methods such as advisory groups, special meetings and committees; and smaller numbers using involving methods (via public meetings) or consulting (via focus groups and surveys), Table 1 and Table 2.*  *The predominant* ***stage of consumer engagement*** *was in development (over 75%), with a third of these [44, 47, 50, 51, 53, 62] (including the four comprehensive health service interventions for healthy birthing) [44, 47, 50, 51] undertaking engagement in both development AND implementation. This seems a logical paired process and again was most common in the community settings, to ensure buy-in and relevance for service delivery in most examples. Only one trial [52] collaborated with consumers at all stages from development through to evaluation. However, the numbers are too small to allow a statistical analysis of the import of this factor on effectiveness.*  *Finally, we considered the* ***characteristics of the consumers*** *in the trials. Again, there was a consistent finding that the consumers were not formally trained in engagement methods prior to the engagement, but the majority received requisite training in the study methodology or intervention to be delivered as part of the engagement process. In three studies [52, 59, 60], health professional participants were reported to receive this same training. Only eight trial consumer cohorts [11, 49, 54, 55, 58, 61, 62, 64] received no training before or during the trial - bringing their lived experience as sufficient expertise for the purposes of the evaluation. This is an important finding for future trials - to consider the engagement of all participants – professionals and consumers – in health care stages across policy, research and services.*  *We found no evidence of adverse effects in engaging with consumers; most trials did not collect data on this aspect. The trials with patient health level data (such as neonatal mortality) [44,50] considered unintended effects in terms of examining the risk ratios for all outcomes between the two groups. None of the included trials addressed possible other adverse effects of consumer engagement, such as tokenism or time and other resource impacts. Seven studies [43,45-47, 50 ,51,53] did consider cost-effectiveness, reporting favourably on the cost per life saved and/or cost of years of life saved in the case of the neonatal care studies, or the cost of the intervention itself, but with no benefits or effectiveness analysis [53].*  ***Strengths and weaknesses of the study***  *By including a strong stakeholder engagement at key stages in the review process we believe we have mitigated against any major biases.*  *Our included trials were wide ranging internationally and across diverse health populations. In particular, we were struck by the number of trials engaging with consumers who were most likely to experience disparity or disadvantage in health services. The settings were predominantly in the community - this seems to lend itself to participatory processes. However, there were some examples of consumer engagement in acute and outpatient settings that augur well for ongoing investigations across the health services spectrum. Positive findings across this range suggests that consumer engagement has a role in many settings, however this does not negate the need for consideration to always be paid to these contextual factors when developing, implementing, monitoring and evaluating consumer engagement processes.*  *Whilst we note the increase in number of more strongly designed (randomised controlled) trials in this important area, most still had a relatively high risk of bias. It should be acknowledged that it can be hard to blind participants – consumers and professionals – in a health care intervention. However, more achievable aspects like adequate randomisation and allocation should be more stringently adhered to, as well as better reporting of outcomes and attrition.*  ***Strengths and weaknesses in relation to other studies***  *Treweek et al [65] published a Cochrane review regarding strategies to improve recruitment to randomised trials. Their key results relating to consumer engagement in recruitment are similar to the results we have found with respect to patient information; in that consumer involvement in developing the content, format and appearance of information leaflets for potential research participants resulted in only a 1% absolute improvement in recruitment , (95% CI: −1% to 3%) [65 p.2]…”* |
| **3^rd^ June 2021**  PONE-D-20-36087R1 Consumer engagement in health care policy, research and services: A systematic review and meta-analysis of methods and effects. PLOS ONE  Dear Dr Wiles,   Thank you for submitting your revised manuscript. During our internal evaluation of the manuscript, we found that there is still significant text overlap between your submission and the following previously published works, particularly in the Introduction and Results sections.  - [https://www.ukcrc.org/wp-content/uploads/2014/03/Piricom+Review+Final+2010.pdf](https://protect-au.mimecast.com/s/wcPDClx15MS9lm3YSgKnvj?domain=ukcrc.org)  - [https://doi.org/10.1016/j.socscimed.2015.03.009](https://protect-au.mimecast.com/s/EURACmO5QMC0q87Qu4eJ4B?domain=doi.org)  - [https://cccrg.cochrane.org/priority-reviews/more-about-our-consumer-engagement-priority-review](https://protect-au.mimecast.com/s/g6PlCnx1rNSyVg1RtP1tfd?domain=cccrg.cochrane.org)  - [https://doi.org/10.1186/s13012-018-0745-6](https://protect-au.mimecast.com/s/6ajGCoV1Q6Com51yUE-DXI?domain=doi.org)  - [https://doi.org/10.1002/14651858.CD004563.pub2](https://protect-au.mimecast.com/s/RHFsCp81QXtlZqJRU33lkH?domain=doi.org)  - [https://www.who.int/evidence/sure/PatientSafetyfullreport10052014.pdf?ua=1](https://protect-au.mimecast.com/s/cahsCq71YMc4vnxMtWa2Zm?domain=who.int)  We would like to make you aware that copying extracts from previous publications, especially outside the methods section, word-for-word is unacceptable, even for works which you may have authored. In addition, the reproduction of text from published reports has implications for the copyright that may apply to the publications.  Please revise the manuscript to rephrase the duplicated text, cite your sources, and provide details as to how the current manuscript advances on previous work. Please note that further consideration is dependent on the submission of a manuscript that addresses these concerns about the overlap in text with published work.  We will carefully review your manuscript upon resubmission, so please ensure that your revision is thorough.  Regards,  Anita Estes PLOS ONE | EMAIL CORRESPONDENCE (9^th^ June 2021):  Many thanks for your response.  We are happy to address this feedback from the Editorial Team, however, need further information in order to make the necessary amendments to our manuscript.  Can you please provide a list (identified by line numbers) of the overlapping manuscript text (i.e. within the Introduction, Methods, Results and/or Discussion) that your evaluations have identified? Our search software, *iThenticate*, is not picking up these references.  Looking forward to hearing from you soon so that we have time to respond and resubmit prior to the due date. |
| **14^th^ May 2021**  PONE-D-20-36087R1  Dear Dr Wiles,  Thank you for submitting your manuscript entitled "Dr Louise Wiles" to PLOS ONE. Your manuscript files have been checked in-house but before we can proceed we need you to address the following issues:  1) Thank you for including your ethics statement on the online submission form: "Human Research Ethics Committee approval was granted from the University of South Australia (protocol number 0000036486) and La Trobe University (approval number S17-013). Written and oral consent was obtained. "  To help ensure that the wording of your manuscript is suitable for publication, would you please also add this statement at the beginning of the Methods section of your manuscript file.  2) Please amend the title either on the online submission form or in your manuscript so that they are identical.  3) Thank you for submitting your revised manuscript. During our internal evaluation of the manuscript, we found that there is still significant text overlap between your submission and the following previously published works, particularly in the Results and Discussion sections.  - <https://doi.org/10.1002/14651858.CD004563.pub2>  - <https://cccrg.cochrane.org/priority-reviews/more-about-our-consumer-engagement-priority-review>  We would like to make you aware that copying extracts from previous publications, especially outside the methods section, word-for-word is unacceptable, even for works which you may have authored. In addition, the reproduction of text from published reports has implications for the copyright that may apply to the publications.  Please revise the manuscript to rephrase the duplicated text, cite your sources, and provide details as to how the current manuscript advances on previous work. Please note that further consideration is dependent on the submission of a manuscript that addresses these concerns about the overlap in text with published work.  We will carefully review your manuscript upon resubmission, so please ensure that your revision is thorough.  Your manuscript has been returned to your account. Please log on to PLOS Editorial Manager at https://www.editorialmanager.com/pone/ to access your manuscript.  Your manuscript can be found in the "Revisions Sent Back to the Author" link under the New Submissions menu. After you have made the changes requested above, please be sure to view and approve the revised PDF after rebuilding the PDF to complete the resubmission process.  Please note that these changes have been requested to comply with submission guidelines and your manuscript will *not* be sent to review until you have fully adhered to our requests. Once your paper has been seen by an Editor we may return it to you for further information or amendments.  We ask that you address this request within 21 days. If you require additional time, please email the journal office. We are happy to grant extensions of up to one month past this due date. If we have not heard from you within 21 days, your manuscript will be withdrawn from Editorial Manager.  Thank you for submitting your work to PLOS ONE.  Kind regards,  Anita Estes  PLOS ONE  __________________________________________________  In compliance with data protection regulations, you may request that we remove your personal registration details at any time. (Use the following URL: https://www.editorialmanager.com/pone/login.asp?a=r). Please contact the publication office if you have any questions. | EMAIL CORRESPONDENCE (17^th^ May 2021):  Thank you for your review and comments.  We have addressed each item of feedback individually (please see below) and marked any necessary changes in the manuscript with the ‘track-changes’ function (please refer uploaded updated copy of our manuscript).   1) We have added this statement to the beginning of the Methods section: “*Human Research Ethics Committee approval was granted from the University of South Australia (protocol number 0000036486) and La Trobe University (approval number S17-013). Written and oral consent was obtained*.” 2) Many thanks – the online submission form title has been updated in line with that of the manuscript (*Consumer engagement in health care policy, research and services: A systematic review and meta-analysis of methods and effects*). |
| **18^th^ March 2021**  PONE-D-20-36087 Consumer engagement in health care policy, research and services: methods and effects PLOS ONE  Dear Dr. Wiles,  Thank you for submitting your manuscript to PLOS ONE. After careful consideration, we feel that it has merit but does not fully meet PLOS ONE’s publication criteria as it currently stands. Therefore, we invite you to submit a revised version of the manuscript that addresses the points raised during the review process.  The reviewer has requested some revisions, in addition to the items raised by the reviewer, please address the following points before more consideration:  Consumer (Or an illustrative term Customer) is a crucial part of any system success, and play a determinant role in policy/ program success and quality. There are a rigorous relationship between customer participation in service delivery and quality improvement.  It appears you needs to a revise your search strategy and search terms to cover all relevant studies.  For example we investigate the role of pregnant women in both quality improvement and assessment activities as customer self-audit. There are many relevant studies using Centering Pregnancy® program to participate women in their own maternity services.  •  Gholipour K, Tabrizi JS, Jafarabadi MA, Iezadi S, Mardi A. Effects of customer self-audit on the quality of maternity care in Tabriz: A cluster-randomized controlled trial. PloS one. 2018 Oct 11;13(10):e0203255.  •  Gholipour K, Tabrizi JS, Asghari Jafarabadi M, Iezadi S, Farshbaf N, Farzam Rahbar F, Afsharniya F. Customer's self-audit to improve the technical quality of maternity care in Tabriz: a community trial. EMHJ-Eastern Mediterranean Health Journal. 2016;22(5):309-17.)    Please submit your revised manuscript by Apr 23 2021 11:59PM. If you will need more time than this to complete your revisions, please reply to this message or contact the journal office at [plosone@plos.org](mailto:plosone@plos.org). When you're ready to submit your revision, log on to [https://www.editorialmanager.com/pone/](https://protect-au.mimecast.com/s/gWfJCwV1mRCZpLOEUVTnam?domain=editorialmanager.com) and select the 'Submissions Needing Revision' folder to locate your manuscript file.  Please include the following items when submitting your revised manuscript:   - A rebuttal letter that responds to each point raised by the academic editor and reviewer(s). You should upload this letter as a separate file labeled 'Response to Reviewers'. - A marked-up copy of your manuscript that highlights changes made to the original version. You should upload this as a separate file labeled 'Revised Manuscript with Track Changes'. - An unmarked version of your revised paper without tracked changes. You should upload this as a separate file labeled 'Manuscript'.   When submitting your revision, we need you to address these additional requirements.  1. Please ensure that your manuscript meets PLOS ONE's style requirements, including those for file naming. The PLOS ONE style templates can be found at:  <https://journals.plos.org/plosone/s/file?id=wjVg/PLOSOne_formatting_sample_main_body.pdf> and  <https://journals.plos.org/plosone/s/file?id=ba62/PLOSOne_formatting_sample_title_authors_affiliations.pdf>  2. Please ensure that you have addressed all items recommended in the PRISMA checklist including identifying the study as a meta-analysis in the title.  3. Thank you for submitting the above manuscript to PLOS ONE. During our internal evaluation of the manuscript, we found significant text overlap between your submission and the following previously published works.  - <https://cccrg.cochrane.org/priority-reviews/more-about-our-consumer-engagement-priority-review>  - <https://doi.org/10.1002/14651858.CD004563.pub2>  - <https://www.who.int/evidence/sure/PatientSafetyfullreport10052014.pdf?ua=1>  We would like to make you aware that copying extracts from previous publications, especially outside the methods section, word-for-word is unacceptable, even for works which you authored. In addition, the reproduction of text from published reports has implications for the copyright that may apply to the publications.  Please revise the manuscript to rephrase the duplicated text, cite your sources, and provide details as to how the current manuscript advances on previous work. Please note that further consideration is dependent on the submission of a manuscript that addresses these concerns about the overlap in text with published work.  We will carefully review your manuscript upon resubmission, so please ensure that your revision is thorough. | Thank you for your review and comments.  We have addressed each item of feedback individually (please see below) and marked any necessary changes in the manuscript with the ‘track-changes’ function (please refer uploaded updated copy of our manuscript).  Please note: Figure 2 had the reference #s updated to align with below changes in the text, but are not tracked.  Thank you for this statement.  The search strategy was developed collaboratively with our stakeholder panel and employed terms and definitions considered relevant and current to consumer engagement in health care policy, research and services. In our review, we focussed on ‘engagement’ in societal decision-making as per the IAP2 Public Participation Spectrum, whereby the engagement is characterised by an inherent social contract.  The two suggested studies (Gholipour et al 2016, Gholipour et al 2018) investigate a model of consumer-centred clinical care where women participate in their own (individual) services, which does not meet our definition of ‘engagement’ at the IASP2 societal decision-making level.  For clarity, we have now moved these definitions from Supplementary Appendix 2 to the main body of the manuscript.  Thank you. We requested and received an extension for our resubmission of May 21 2021.  Our resubmission includes:   - A rebuttal letter (this document) - A marked-up copy of the manuscript - An unmarked version of our revised paper   Thank you for clarifying the naming protocols for the files. The files have been named as requested. Thank you for the example links. Formatting changes have been made (and tracked) to comply with the style requirements.  Many thanks – we have the addressed the PRISMA recommended checklist items by making the following changes:  1. Title changed to: ***Consumer engagement in health care policy, research and services: A systematic review and metanalysis of methods and effects*.**  2. The page numbers in the PRISMA checklist have been updated to reflect the changes made, including additional information.  3. We are concerned at these significant text overlaps.  The first (Cochrane) website is advertising the commencement of our current review and contains text overlap in authors, and the Prospero links to the protocol - neither of which we can avoid. This Cochrane website is out of date and we will request it be removed.  The second document is the precursor Cochrane review to our current review and the text overlap is the listing of search strategies and databases which again has been replicated unavoidably. We have clearly cited our review is an update of this review.  The third report is a Patient Safety report – we have checked the text overlap using *ithenticate* and found there is minimal overlap with Cochrane methods terminology.  We remain concerned about your findings and would welcome further guidance if the above does not adequately address your concerns.  Our *ithenticate* report is attached – the similarities are within our preprint and (erroneously posted in Research Square as a pre-print) and in cited text, references etc. |
| **REVIEWER 1** |  |
| Reviewed for PLoS One  Feb 18, 2021 | Many thanks. |
| These authors evaluate controlled trials that examine the potential benefits of engaging  consumers in the development of health care services, policy or research. Given the increased  focus on including diverse voices in health care policy and systems, this review is timely. | Thank you for this feedback. |
| Overall this review is sound and useful. The authors use rigorous criteria for the most part,  including registering their trial and using a coherent and sound matrix for classifying  interventions and outcomes. I would like to see an Introduction that better framed the  contribution, particularly in light of recent efforts to include a diversity of voices and  communities in health care versus the extra resource cost of doing so. I also note a potential  problem in the meta-analysis across 4 studies in the Results section; authors need to make sure  they are not comparing odds ratios to each other across studies. | We appreciate this feedback and have specifically addressed these points below. |
| There are several other minor changes that I noted, which I believe will make the review  stronger and more clear.  All suggestions are meant to help the authors as they refine this timely work. | Thank you for the constructive feedback which we will use to refine and improve this work. |
| ***Major Revisions*** |  |
| Authors need to do a better job of explaining the motivation for their work including their  definition of consumer engagement. Consumer engagement, at least in the United States,  often means engagement and responsibility for one’s own medical decisions. Authors note in  Appendix S2 that their definition of consumer engagement does not encompass one’s own  medical decisions, rather they are looking at role of consumers in societal medical decision making. This definition needs to be stated in Introduction, not in Appendix. | We agree that the Introduction would benefit from a clearer definition of consumer engagement in this review, and have included this in the last paragraph (lines 135-138):  *“In our review, we aim to report on: (a) the methods of consumer engagement strategies used in societal decisions on health care policy, research and services (according to the IAP2 Public Participation Spectrum); and, (b) their effects on the people involved in the engagement, on the research/policy/health care service, as well as process outcomes.”* |
| Further, on this same point, authors need to reframe their Introduction to better outline what they hope to learn from this review. Lines 97-99, for example, note that there is already evidence of consumer engagement leading to more accessible and acceptable health services--- so why is that studied? The authors also note their main intention is to study “effects”—effects on what? This term is so vague that it is difficult to determine what they are hoping to learn. I was intrigued by the last sentence of the first paragraph, that mentions that intentions around consumer engagement might not live up to the reality of what it is able to achieve, but could not find a description of what exactly those intentions are. (The sentence just prior mentions actual evidence of benefit.) Better organization and framing here would help focus the review. | We have addressed this feedback in the following ways:   (1) detailing the “effects” of interest to the review; lines 135-138:   *“In our review, we aim to report on: (a) the methods of consumer engagement strategies used in societal decisions on health care policy, research and services (according to the IAP2 Public Participation Spectrum); and, (b) their effects on the people involved in the engagement, on the research/policy/health care service, as well as process outcomes.”*   (2) Explaining the need to study the effects of consumer engagement in health services (despite existing Level 1 evidence of consumer engagement leading to more accessible and acceptable health services, which comes from a systematic review published in 2002), and clarifying the ‘intentions’ of consumer engagement (i.e. reframing as intended benefits); lines 99-102:   *“That said, there is a lack of contemporary research that reliably or systematically investigates whether consumer engagement achieves these intended benefits and if so, which methods of consumer engagement are most effective, and how these effects might be measured.”* |
| Lines 250-251: Notes that they contacted study authors in some cases. How often did authors  have to contact study authors and what questions were not included in manuscripts? In results, (lines 418-421) authors report that overall risk of bias was high confounded by poor reporting especially in older studies. More explanation about whether bias risk was due to study design or poor reporting, and how often author contact helped, would help validity of this study. | We have addressed this feedback by:   - Providing details of the number/frequency and success of author contact in the Results section; lines 433-438:   *“Study authors (n=4) were contacted via email (up to a maximum of three occasions over a two-month period) to clarify: (i) study completion dates (for published study protocols and pilot/foundational studies that indicated the main study was still in progress), and (ii) details of the consumer engagement strategies (especially how they were developed) to determine if they met our eligibility criteria. Author contact for the latter was not especially helpful as methodological ambiguity most often arose from older studies, the authors of which typically did not reply.”*   - Outlining the differences in risk of bias ratings between the earlier/more recent studies: *“Overall the risk of bias in the included studies was high, confounded by poor (unclear) reporting particularly in the earlier studies as noted in the 2006 and 2009 reviews; on average, there were 6.3 items with unclear risk of bias ratings for the 7 studies published between 1997 and 2007), compared with 2.3 items for those (n=16) from 2008 onwards.”* |
| Results: Lines 444-445: Individual odds ratios across studies are not able to be compared given the different modeling methods and covariates used in individual studies. Thus, the authors cannot conclude that heterogeneity in individual odds ratios represents actual heterogeneity across outcomes. (See Norton, Dowd, Maciejewski; JAMA. 2018;320(1):84-85.) | We agree individual odds ratios across studies cannot be compared. In our meta-analysis we used the originally reported number of events (and total number in each group) to calculate one meta Odds ratio for each outcome. Our protocol allowed for the calculation of risk ratios – we can provide these if the reviewers prefer. Our statistical advice is that both are equally relevant in this context. |
| Figures 3/4: It appears odds ratios are being compared across studies in both these figures. It does not appear that odds ratios were compared to generate the overall OR of 0.84 or 1.10, but these comparisons of OR across studies is not valid as explained in citation above. This figure should be removed or modified so as to avoid such comparisons. | Please see above.  As observed, we have not used odds ratios to generate the meta Odds Ratio for either outcome.  The figures are standard Forest plots using the best practice REVMAN tool and methods as described in the Cochrane Handbook.  We have modified the text to ensure readers understand the figures, and that we are not comparing OR across studies (lines 531-538):  *“For the outcomes of neonatal mortality,*  *we extracted data for 83,925 births, with a total of 1,028 deaths for the intervention group and 1,282 for the control group; this gave a meta odds ratio of 0.8 in favour of the intervention group (95% CI 444 0.77, 0.91; p<0.0001) (Fig 3)* *(or Risk Ratio of 0.84; 95% CI 0.77, 0.91; p<0.0001). The heterogeneity (as assessed by the I2) was high at 72% and mostly explained* *by the large range of frequencies of event. The second outcome (maternal deaths) that afforded meta-analysis returned a non-significant finding from the same four studies [38, 41, 44, 45] and same number of births with 80 maternal deaths in the intervention group versus 78 in the control (OR of 1.10, 95% CI 0.81,1.51; p=0.54). (Fig 4).* *A risk ratio analysis revealed the same results.* |
| ***Minor Revisions*** |  |
| Lines 138-146: these lines contain sentence fragments and don’t seem to fit well into the flow of the Introduction. Either cut or set up better in preceding text. | We have reworded as (lines 141-143):  *“The primary objective of the review was to assess the effects of consumer engagement on health care policy, research and services. Secondary objectives were to explore whether differences between studies might explain any differences between the effects. We were specifically interested in differences in the:*   - *methods (levels) of consumer engagement (e.g. fact sheets, focus groups, patient advisory committees representing the levels of inform, consult, involve, collaborate or empower);* - *stages (i.e. development, implementation, monitoring, evaluation) of health care policy, research and services in which consumers are engaged; and* - *characteristics of consumer or professional participants (e.g. background, experience or training in consumer engagement).”* |
| Methods: Type of studies. Define what you mean by each of these study types, especially your definition of quasi-RCT | We adopted, and have now referenced, the Cochrane Collaboration Handbook definitions for study type (lines 249-250). |
| Lines 490-498: This section, “Patient Information” is equivocal in its conclusions, and I think it could be written more definitively. I realized there are only two studies, but not whether outcomes all went the same way, whether there were differences in outcomes between studies, etc. Certainly noting limitation of those conclusions is warranted, but as written now this section is very vague and it’s hard to draw anything from it. | Thank you for this feedback; we have reworded this section and included more of the suggested detail:  “…*Patient information*  *Two trials evaluated products (patient information leaflets about endoscopic procedures [49] and post-operative patient-controlled analgesia (PCA) [55]) which were developed following consumer consultation [49, 55]. The leaflets were compared with patient information developed without consumer consultation. Based on these two trials [49, 55] evidence is equivocal for that consumer consultation prior to developing patient information material; while the CE material resulted in better anxiety outcomes for endoscopy patients in one study (no effect for satisfaction) [49], the other showed that despite the CE material presenting information more clearly and improving patient knowledge of PCA, there was no effect for worry about PCA-use [55]. Both studies had unclear risk for all biases (i.e. not stated within methodology) except for low risk of selection bias (use of random allocation) [55], and high risk for attrition (not all outcome data reported, no reasons given) and selective reporting bias [55] (Figure 2).”* |
| Lines 497-498: “Both studies had some unclear risk of bias issues.” This sentence and idea need more explanation. What do you mean here? The issues were unclear or the risk of bias was unclear? Unclear how? | We have addressed this as part of the preceding item response (please see above). |
| Line 517: Sentence beginning “Boivin et al involved consumers….” There is a word missing in this sentence. | Thank you – we have reinstated the missing word *“consumer”* in this sentence. |
| Lines 525-527: “There is also some evidence of consumers contributing positively to identifying need and developing mental health services directions, as well as supporting decision-making processes in health.” Where is support for this sentence? Is it from Carmen et al study? If so, needs to be made more explicit what outcomes/policies were measured in this study. If not, need citation and possibly study description. | Thank you; we have reworded this passage to better reflect our meaning and have included additional detail about the outcomes measured:  “…. *Based on these studies there is some evidence that consumers may have a role in identifying a broader range of health care priorities that are complementary to those identified by professionals [39, 48]. In addition to supporting decision-making processes in health (reflected in public deliberation increasing participants’ knowledge of and attitudes towards the role of medical evidence) [58], there is also some evidence of consumers contributing positively to identifying need and developing mental health service directions [47]. The fourth policy-based trial [47] collaborated with consumers with previous mental health issues in identification, planning, development (and implementation) of community-based services. The services developed with consumer collaboration were superior to services developed with no consumer engagement in all measured items (mental health scores, service use and so forth), except employment and medication use…*” |
| Lines 590-591: given that the neonatal/maternal mortality is one of the primary results, and the only one developed with statistical meta-analysis, more explanation of how the interventions differed between the consumer engagement interventions vs control arms would help. In these interventions, what were some key differences that might have led to better outcomes for the treated group? | We have addressed this below – with thanks. |
| Lines 623-629: I see some more explanation here, but descriptions are very general (e.g.  “sustainable community development”. More specifics, if you have them, about how  these interventions differed would be very helpful for those reading this review to get  practical advice on how to develop such interventions. | Thank you – we have included more specific information about the CE interventions:  *“…Other key characteristics were that the local community (i.e. women’s groups, community leaders and member, locally recruited facilitators) were embedded in every phase of the CE program; in developing its content, its implementation plan, and its delivery…”*  If desired and deemed to be helpful for the readership, we would be happy to present summaries of the characteristics of included studies as ‘Supporting information’ in the form of an Appendix. |
| Lines 678-684: Look again at this section. The pulled quote from the Treweek review is  confusing and I had a hard time figuring out how it related to these authors’ results. | Thank you for this suggestion, we have now reworded this section:  *“…Treweek et al [59] published a Cochrane review regarding strategies to improve recruitment to randomised trials. Their key results relating to consumer engagement in recruitment are similar to the results we have found with respect to patient information; in that consumer involvement in developing the content, format and appearance of information leaflets for potential research participants made little or no difference to recruitment (absolute improvement was 1%, 95% CI: −1% to 3%).…*” |
| Lines 693-695: “Health professional can be confident that engaging consumers can have a  positive effect in health care policy, research and services, however there are not standard  metrics to guide evaluation of this effect.” This sentence needs to be changed as it does not  have empirical support. If there are no metrics, how do we know effect is positive? Consumer  engagement takes additional resources that could be directed to other productive ends. The  best use of this section is to help policymakers and clinicians understand the most productive  use of consumer engagement, as we know it from existing controlled studies. | Thank you for this comment.  While we agree with the tenet of this feedback, we would like to highlight that metrics do exist for evaluation; it is the lack of standard metrics that limit the comparison of different methods across health care settings. We have reworded this section and tempered our language accordingly:  *“…Engaging consumers can have a positive effect in health care policy, research and services, however there are no standard metrics to guide and benchmark evaluation of this effect across settings and contexts…”*  The existing subsequent passage of text goes on to help policymakers and clinicians understand the most productive use of consumer engagement (no further change has been made).  *“…The factors that underpin successful consumer engagement can be hypothesised from the included trials as involving consumer empowerment processes, in the development and implementation phases; there is a lack of evidence arising from the trials regarding the effect of other elements of engagement (inform, consult, involve, collaborate and empower) across all phases. Our recommendation is to use a tailored approach to consumer engagement with an IAP2 level that is as high and appropriate to the goals and promises to the public, and embedded evaluation…”* |
| Figure 1: In final box of included studies, what is meaning of “plus 5 awaiting classification and 1  ongoing study”. Are these included in analysis? I don’t think they are but notes in figure and  explanation of why they are in the box with final included studies is warranted. | Figure 1 has been clarified. |
| **REVIEWER 2** |  |
| This is a thorough and careful job, and this work will be of interest to everyone who wishes to promote patient engagement in health services, policy and research. I am recommending acceptance because I do not feel that I need to see it again. I do have a couple of comments. | Thank you. |
| First, it is surprising that the authors report no specific funding for such a substantial effort. Surely somebody must have been paying salaries for this. Also, the idea that you involved patient advocates and other stakeholders in the work without providing any compensation seems contrary to the spirit of the thing. I think readers would want to know how this was resourced. | We have added the following: *The University of South Australia provided salaries for the author SH, and via grant funding for LW, JL and AW. We are very grateful for the volunteer work of DK and the stakeholder group*. |
| It is rather odd to see repeated use of the conditional in the methods. If it turned out that the nature of the available publications did not allow you to use a certain method you can just say that. | We acknowledge that the conditional can sound somewhat awkward however it is the style used to ensure a match with the protocol. |
| On the whole, with the exception of neonatal mortality, the evidence for most of the outcomes seems quite weak. This is not only because of risk of bias, but also because in many cases the investigators looked at multiple outcomes and found evidence for some but not others. The positive findings often pertained to intermediate outcomes and not to more downstream outcomes, as well. While you do generally acknowledge the need for more evidence and you are restrained in endorsing conclusions, I still have an overall feeling that you might be overselling the findings just a bit. Maybe that's just me. Anyway I appreciated this and I think it's a worthy contribution | Please see response to reviewer 1 to correct the impression of overstating. Thank you for your comments on the worth of this contribution. |
